# Ligand-Based Discovery of a Small Molecule as Inhibitor of α-Synuclein Amyloid Formation

**DOI:** 10.3390/ijms232314844

**Published:** 2022-11-27

**Authors:** Laura De Luca, Serena Vittorio, Samuel Peña-Díaz, Giovanna Pitasi, Marc Fornt-Suñé, Federica Bucolo, Salvador Ventura, Rosaria Gitto

**Affiliations:** 1 Department of Chemical, Biological, Pharmaceutical and Environmental Sciences, University of Messina, Viale F. Stagno D’Alcontres 31, I-98166 Messina, Italy; 2 Institut de Biotecnologia i Biomedicina, Universitat Autonoma de Barcelona, 08193 Bellaterra, Spain; 3 Departament de Bioquimica i Biologia Molecular, Universitat Autonoma de Barcelona, 08193 Bellaterra, Spain; 4 ICREA, Passeig Lluis Companys 23, 08010 Barcelona, Spain

**Keywords:** alpha-synuclein, Parkinson’s disease, small molecule, virtual screening, binding site prediction, Th-T fluorescence assay

## Abstract

α-Synuclein (α-Syn) aggregates are implicated in Parkinson’s disease (PD), so inhibitors of α-Syn aggregation have been intensively explored. It has been demonstrated that small molecules might be able to reduce α-Syn aggregation in fibrils, thus exerting neuroprotective effects in models of PD. To expand our knowledge about the structural requirements for blocking the recognition process into the oligomeric assembly of α-Syn aggregates, we performed a ligand-based virtual screening procedure using two well-known α-Syn aggregation inhibitors, SynuClean-D and ZPD-2, as query compounds. A collection of thirty-four compounds bearing distinct chemical functionalities and mutual chemical features were studied in a Th-T fluorescence test, thus identifying 5-(2,6-dinitro-4-(trifluoromethyl)benzyl)-1-methyl-1H-tetrazole (named MeSC-04) as a potent α-Syn amyloid formation inhibitor that demonstrated similar behavior when compared to SynuClean-D in the thioflavin-T-monitored kinetic assays, with both molecules reducing the number and size of amyloid fibrils, as evidenced by electron microscopy. Molecular modeling studies suggested the binding mode of MeSC-04 through the identification of putative druggable pockets on α-syn fibrils and a subsequent consensus docking methodology. Overall, this work could furnish new insights in the development of α-Syn amyloid inhibitors from synthetic sources.

## 1. Introduction

Parkinson’s disease (PD) is a chronic and progressive neurodegenerative process that is commonly related to the loss of dopaminergic neurons in the substantia nigra pars com-pacta (SNpc). It has been demonstrated that PD is characterized by an abnormal intraneuronal accumulation of α-synuclein (α-Syn) aggregates called Lewy bodies, which are considered to be responsible for the progression to tremors, rigidity, posture instability, akinesia, and cognitive impairment [1]. The native monomeric form of α-Syn is intrinsically disordered in solution; it polymerizes into oligomers that form higher-order species and fibrils that accumulate in Lewy bodies and Lewy neurites, leading to synucleinopathy [2]. The α-Syn monomers are water-soluble and nontoxic, whereas misfolded α-Syn accumulates as insoluble fibrils that aggregate through the formation of soluble oligomeric intermediates, generating insoluble forms that are toxic to neurons [3]. The α-Syn monomer is a small protein containing three domains: an N-terminal domain (1–60), a central nonamyloid component (NAC) domain (61–95), and a C-terminal domain (96–140). The N-terminal domain forms an amphipathic lysine-rich α-helix that is responsible for the ability of α-Syn to associate with vesicles and membranes. The central NAC domain of α-Syn controls misfolding into β-sheet-rich amyloid aggregates. Finally, the polar C-terminal domain contains negatively charged serine and tyrosine residues that can be phosphorylated, regulating the membrane binding, aggregation, and toxicity of α-Syn [3]. It has been demonstrated that increases or decreases in α-Syn levels are implicated in regulating neurotransmitter release, synaptic activity, and plasticity in physiological conditions. However, α-Syn aggregates have been linked to the progression and spread of pathological adducts that correspond with the clinical progression of PD. Therefore, it is presumed that therapeutic interventions modulating α-Syn misfolding may prevent or delay the progression of PD to the disabling motor symptoms and eventually cognitive impairment linked to neuronal synucleinopathies [4]. 

**Figure 1:**
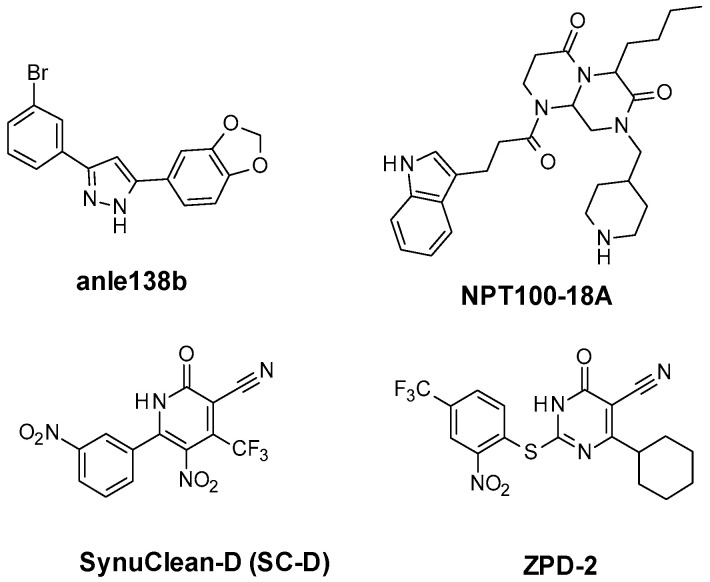
Chemical structures of Anle138b, NPT100-18A, SynuClean-D (also known as SC-D), and ZPD-2, well-known inhibitors of α-synuclein aggregation.

Therefore, the inhibition of these pathogenic α-Syn aggregates could offer opportunities in the treatment of PD in early states before the occurrence of irreversible neurodegenerative processes. It has been established that small molecules could interfere with the interaction of β-sheet surfaces and misfolded α-Syn oligomers, thus preventing their aggregations. The misfolded proteins are generally considered “low druggable” targets, as they lack specific ligand-binding sites that efficiently establish strong binding with inhibitors or modulators. However, there are recently developed synthetic and natural small molecules that inhibit α-Syn aggregation and fibril formation in cell-based assays [5,6,7,8,9,10,11,12,13,14]. These therapeutics are generally defined as disaggregators and might be characterized by a distinct mode of action. Among them, several compounds (Figure 1) have entered clinical trials: 5-(1,3-benzodioxol-5-yl)-3-(3-bromophenyl)-1*H*-pyrazole [15,16] (Anleb138b) proved to inhibit α-Syn oligomer formation, whereas 1-(3-(1*H*-indol-3-yl)propanoyl)-6-butyl-8-(piperidin-4-ylmethyl)hexahydro-4*H*-pyrazino [1,2-a]pyrimidine-4,7(6H)-dione (NPT100-18A) [11] was demonstrated to interfere with α-Syn aggregation by displacing the protein from membranes. Moreover, the thioflavin-T assay (Th-T) identified 5-nitro-6-(3-nitrophenyl)-2-oxo-4-(trifluoromethyl)-1*H*-pyridine-3-carbonitrile (SynuClean-D, also known as SC-D, Figure 1) [13] and 4-cyclohexyl-2-((2-nitro-4-(trifluoromethyl)phenyl)thio)-6-oxo-1,6-dihydropyrimidine-5-carbonitrile (ZPD-2, Figure 1) [17] as new α-Syn aggregation inhibitors.

Successively, exciting outcomes from the experimental in vivo and in vitro data of the 2-pyridone-based SynuClean-D and 4-pyrimidinone-based ZPD-2 prompted researchers to develop new analogs that demonstrated promising biological properties [8,9,10]. These efforts revealed that in some cases a single aromatic ring decorated with suitable very simple molecular moieties constitutes the magic player to reduce α-Syn aggregation in in vitro models [9].

## 2. Results and Discussion

Here, we report a computational and experimental screening to identify new potential inhibitors of α-Syn aggregation that are structurally related to well-known active compounds. Moreover, we analyzed their plausible binding pocket located on the NAS domain.

### 2.1. Similarity-Based Virtual Screening (VS)

To find new chemical entities, we employed the SwissSimilarity web tool to perform a similarity-based virtual screening (VS) procedure, which is a method based on the well-established assumption that similar molecules might be prone to produce similar biological effects. The free-of-charge SwissSimilarity platform (http://www.swisssimilarity.ch, accessed on 1 February 2021) can perform a ligand-based virtual screening (LBVS) on large libraries of commercially available molecules, including approved drugs as well as bioactive compounds. The selection of the VS candidates is generally performed through the application of bidimensional and three-dimensional search methods, starting from a specific query compound following the screening procedure information provided by the SIB Swiss institute of Bioinformatics [18,19]. In this work, the two inhibitors SC-D and ZPD-2 were used as reference structures (Figure 1), while the SPECS library, composed of 326,000 commercially available small molecules, was selected for the searching procedure. To estimate the similarity between the query molecules and the compounds contained in the screening library, we applied three different methods: FP2 fingerprints, Electroshape-5D, and spectrophores. The first one is a 2D similarity method that encodes linear molecular fragment paths of up to seven atoms into a 1024 bit string [20]. Instead, ElectroShape [21] and spectrophores [22] are 3D similarity methods based on molecular shape complementarity; these methods do not require geometry alignment, making the VS process faster. In both methods, the molecular shape is described by 3D molecular properties whose information is compressed into vectors [19]. The three methods were independently applied for each query molecule, and the number of compounds obtained from each approach is reported in Table 1.

**Figure 2:**
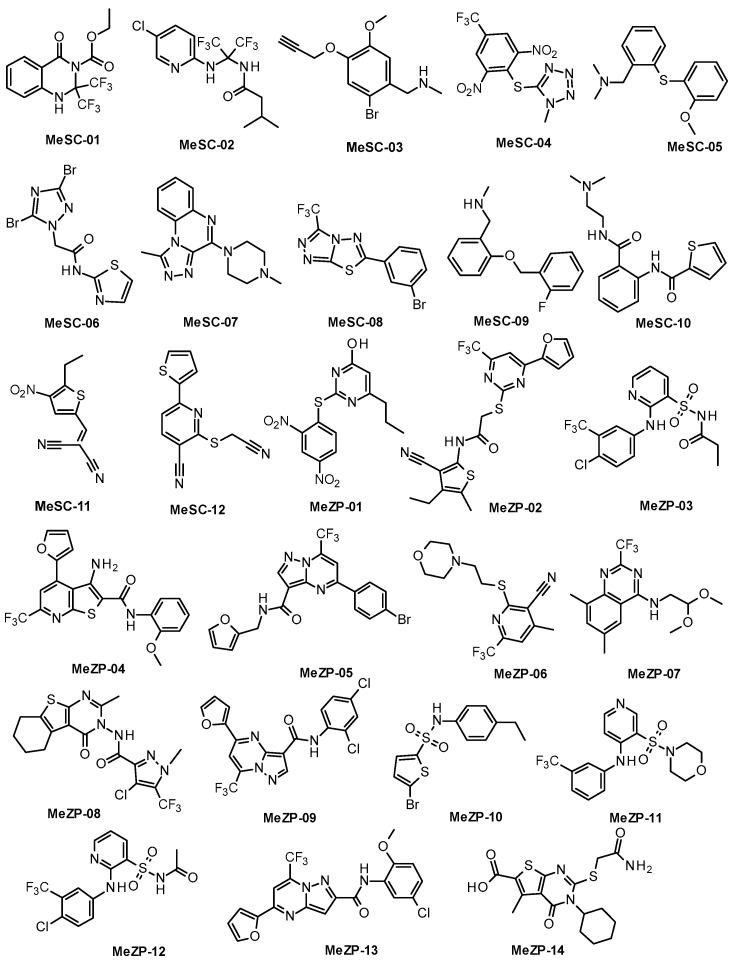
Chemical structures of compounds resulting from the VS employing SC-D (compounds MeSC-01–MeSC-12) and ZPD-2 (compounds MeZP-01–MeZP-22) as query compounds.

By using SC-D as a query, the application of FP2 fingerprints led to the identification of three hits, whereas two hits were obtained from the ZPD-2 query compound. In turn, the Electroshape method yielded thirty-three compounds whose Manhattan distance (MD) values varied from 0.861 to 0.707 in the case of the SC-D query compound. Instead, four-hundred hits, with MD scores ranging between 0.930 and 0.831, were obtained with the same approach starting from the inhibitor ZPD-2. In this case, we further filtered off the resulting hits according to the similarity score by selecting one hundred and seventy-eight molecules whose MD values were below 0.855. Finally, 400 hits were identified through the spectrophore method for both compounds. The obtained hits fell in a similar range of MD values that were between 0.833 and 0.764 when SC-D was used as reference and between 0.819 and 0.754 when ZPD-2 was employed. Therefore, we applied the same MD threshold value of 0.785 as a prefilter, thus obtaining 39 compounds from ZPD-2 and 108 hits from SC-D. Overall, 363 molecules were chosen from the LBVS runs for further analysis. Specifically, we discarded the compounds with stereocenters or E/Z bonds whose configuration or geometry was not defined. Concerning the derivatives possessing similar scaffolds, we selected those compounds characterized by the highest similarity scores. Finally, the remaining molecules were filtered according to (i) their drug-like properties, measured by the Lipinsky rule of five; (ii) the absence of PAINS, calculated by the SwissADME platform (http://www.swisssimilarity.ch, accessed on 1 February 2021); and (iii) the availability from commercial suppliers. The application of these filters led to the final selection of thirty-four compounds collected in Figure 2; among them, the first subset of twelve molecules (MeSC-01–MeSC-12) was retrieved using SC-D as a query molecule, whereas the second subset of twenty-two molecules (MeZP-01–MeZP-22) was obtained from ZPD-2.

### 2.2. Thioflavin-T Fluorescence Test

**Figure 3:**
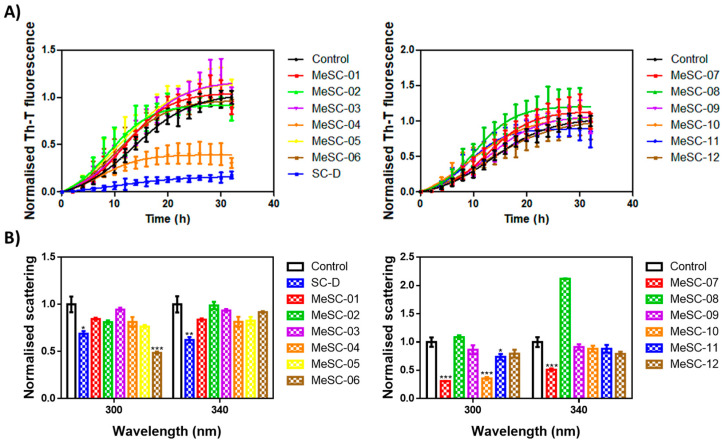
In vitro inhibitory characterization of compounds MeSC-01 to MeSC-12. (**A**) Aggregation kinetics of α-Syn in the absence (black) or presence of 100 µM concentrations of the different compounds (colored) compared to SC-D (blue, left panel). Th-T fluorescence is plotted as a function of time. (**B**) Light scattering measurements at 300 and 340 nm of end-point aggregates in the absence (black) or presence of 100 µM concentrations of the different compounds (colored) compared to SC-D (blue, left panel). The statistical significance was determined by a one-way ANOVA test with Dunnett’s multiple comparison (* *p* < 0.05; ** *p* < 0.01; *** *p* < 0.001). For the SC-D and MeSC-04 compounds, the Th-T levels at the final point were statistically significant (*p* < 0.0001). Th-T fluorescence and light scattering are plotted as normalized means; error bars represent SEs of mean values for N ≥ 3 in each experiment.

**Figure 4:**
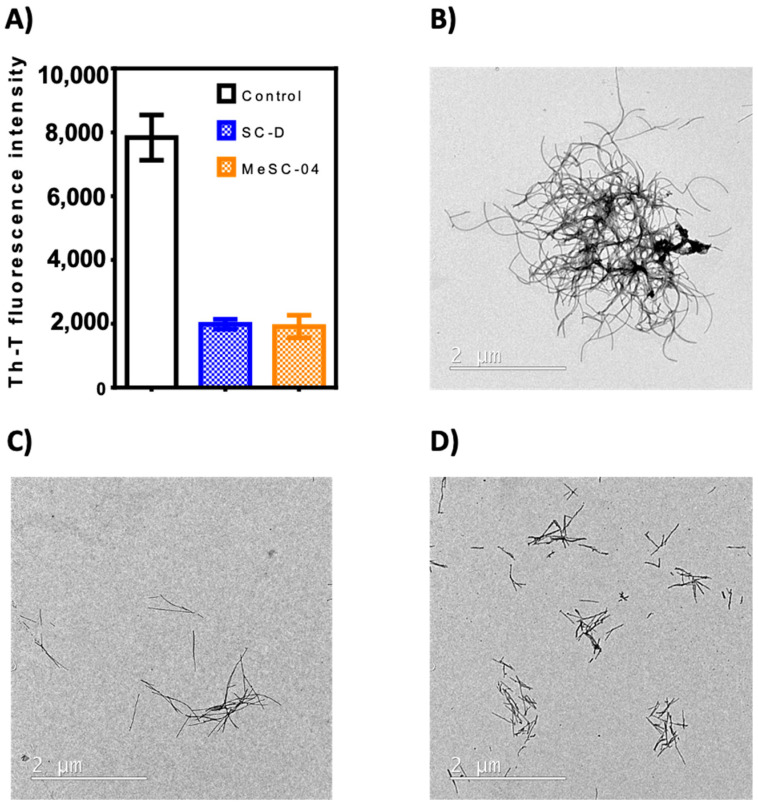
In vitro inhibitory characterization of compounds SC-D and MeSC-04. (**A**) End-point Th-T fluorescence signal after 48 h of aggregation of α-Syn in the absence (white) or presence of 100 µM concentrations of compounds SC-D (blue) and MeSC-04 (orange). (**B**–**D**) Representative transmission electron microscopy images of end-point aggregates in the absence (**B**) or presence of 100 µM concentrations of compounds SC-D (**C**) and MeSC-04 (**D**). SC-D was used as reference for the inhibition of α-Syn aggregation. Each experiment was repeated at least in triplicate. A one-way ANOVA test with Dunnett’s multiple comparison was performed to analyze the statistical significance.

The thirty-four candidates were purchased from a SPECS vendor and tested in the thioflavin-T fluorescence test as a preliminary assay to measure their ability to reduce α-Syn aggregation, as already reported for SC-D and ZPD-2 [8,9,10]. To evaluate the inhibitory potential of the selected compounds, we monitored the increase in thioflavin-T (Th-T) fluorescence emission during the aggregation kinetics of 70 µM α-Syn in the absence or presence of a 100 µM concentration of the molecules. SC-D was used as a reference compound. MeSC-04 emerged as the best candidate, with a Th-T fluorescence emission reduction of 69% (*p*< 0.0001) (Figure 3A). This evidence was confirmed by titration at endpoint measurements at different doses of MeSC-04, as displayed in Figure S3 (see Supporting Material), as it was effective at substoichometric concentrations and, relative to α-Syn MeSC-04, did not reduce light scattering significantly, which may suggest that it diverts the aggregation reaction towards the formation of nonamyloid assemblies (Figure 3B). To discard this possibility, we performed new α-Syn aggregation assays in the absence and presence of SC-D, used as a positive control, and MeSC-04. Then, we imaged the resulting protein solutions after 48 h of incubation by transmission electron microscopy (TEM). As can be observed in Figure 4, the reduction in Th-T fluorescence by both SC-D and MeSC-04 was accompanied by a significant decrease in the number and size of observed fibrils relative to the untreated α-Syn solution. This confirms the antiamyloid character of both molecules.

**Figure 5:**
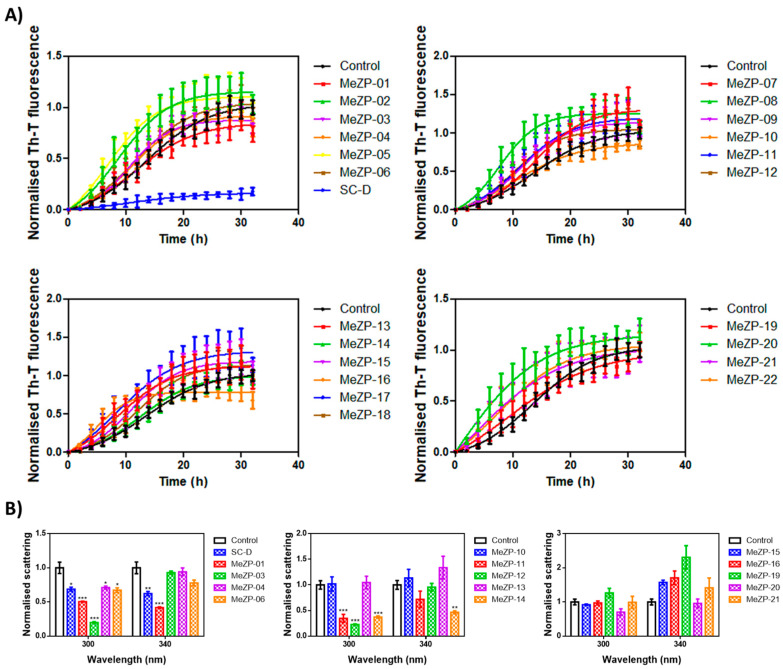
In vitro inhibitory characterization of compounds MeZP-01 to MeZP-22. (**A**) Aggregation kinetics of α-Syn in the absence (black) or presence of 100 µM concentrations of the different compounds (colored). SC-D was used as a reference (blue, left panel). Th-T fluorescence is plotted as a function of time. (**B**) Light scattering measurements at 300 and 340 nm of end-point aggregates in the absence (black) or presence of 100 µM concentrations of the different compounds (colored). SC-D was used as a reference (blue, left panel). The statistical significance was determined by a one-way ANOVA test with Dunnett’s multiple comparison (* *p* < 0.05; ** *p* < 0.01; *** *p* < 0.001). Th-T fluorescence and light scattering are plotted as normalized means; error bars represent SEs of mean values for N ≥ 3 in each experiment.

The MeZP derivatives did not show a significant reduction in α-Syn amyloid formation (Figure 5A). The best candidates, MeZP-01 and MeZP-16, reduced the final Th-T fluorescence by 26 and 32%, respectively (Figure 5A). Regarding light scattering, among both compounds, only MeZP-01 significantly decreased the signal, by around 50 and 58% for 300 and 340 nm, respectively (Figure 5B). The lack of activity of MeZP-16 in the light scattering assay may indicate the formation of alternative structures such as amorphous aggregates.

All in all, the in vitro screening exercise highlighted MeSC-04 (chemical name: 5-(2,6-dinitro-4-(trifluoromethyl)benzyl)-1-methyl-1*H*-tetrazole; its structural characterization is provided in Figure S2) as the best molecule, as it was proven to be active, as previously found for SC-D.

### 2.3. Computational Studies to Predict the Binding Site

**Figure 6:**
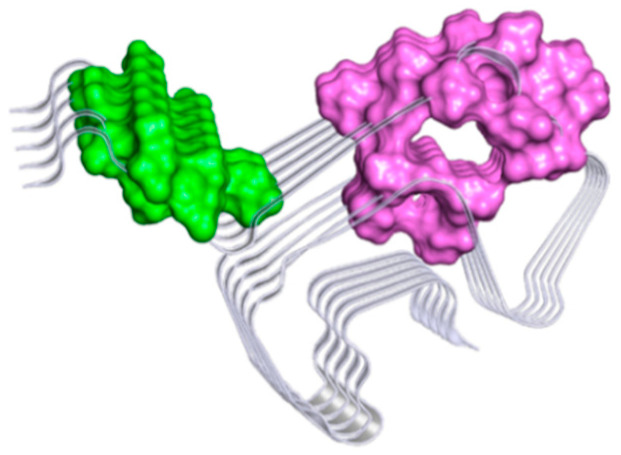
Ensemble consensus sites highlighted as different colored surfaces: site A (colored in magenta) and site B (colored in green). The image was prepared by the Pymol program [25].

To study the putative binding mode of MeSC-04, a two-step computational study was performed as follows: (i) a ligand pocket analysis and (ii) molecular docking simulations. Several computer programs capable of identifying binding pockets or allosteric sites have been developed and are classified according to the type of algorithm used (docking-based, grid-based, and geometry-based algorithms). In this work, three different programs, fPocket, SiteMap, and FTMap, were used to evaluate the druggability of α-Syn fibrils for two three-dimensional structures that are available in the RCSB PDB databases (codes 2N0A [23] and 6FLT [24]). A detailed description of the binding pocket identification procedure is reported in the Material and Methods. By combining the output from the three tools, several key amino acid residues were identified as “hot spots” and clustered to obtain two ensemble consensus sites, namely site A (colored in green) and site B (colored in magenta), as displayed in Figure 6.

Table 2 describes all residues forming the two detected druggable binding sites. The obtained results were compared with the data already reported in the literature [13,26,27,28] for other inhibitors of aggregation.

In more detail, the comparative analysis showed that site A resulted in good agreement with the previously defined binding site of SC-D [13]. It was demonstrated that the amino acids Ala53, Val55, Thr59, Glu61, Thr72, and Gly73 were responsible for the interaction [13]. In addition, the amino acid residues Thr54, Ala56, Thr59, Glu61, Gly73, Val74, and Thr75 of chain B-C-D of site A were reported as key residues for hydrophobic and π-alkyl interactions between the complex sulfated polysaccharide named ulvan and α-Syn in a recent study by Wenqian Wang [26]. In contrast, site B was highlighted and identified as site 3/13 (Lys43, Lys45, Val48, and His50) in a study based on a search for different putative binding sites on α-Syn fibrils by the blind molecular docking of small molecules performed by Chia-Ju Hsieh et al. [27].

Furthermore, Jiang Bian et al. have studied the binding of some derivatives of styrylaniline, and SAR analysis, further rationally clarified by molecular docking studies, revealed that H-bonds and cation–pi interactions with Lys43, Lys45, and Lys58 of chain B-C-D are crucial for binding. All described residues are part of our site B [28]. Overall, the outcomes of our computational study were in good agreement with other in silico binding sites analyses performed on α-Syn fibrils.

Prompted by these results, the binding mode of the active compound MeSC-04 was investigated. To select one off the two fibril structures as a target for docking studies, we evaluated their quality with a Ramachandran plot through PROCHECK [29]. The evaluation data indicated that 2N0A was the best structure, with 85.1% of residues in the most favored region (see Table S1 in the Supplementary Material).

To probe the molecular interactions between MeSC-04 and α-Syn fibrils, three different programs, Autodock V4.2.6 [30], Glide-v9.3 [31,32], and Gold 2020.3.0 [33], were used to perform docking studies on both previously reported putative binding sites (A and B).

**Figure 7:**
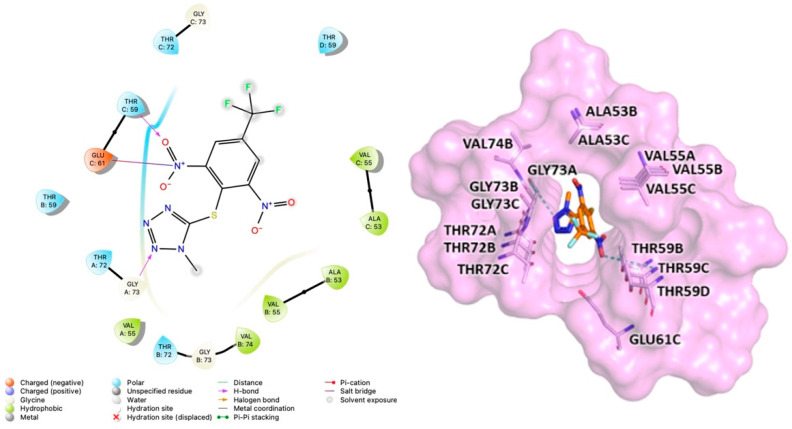
Binding site of α-Syn identified through docking studies for compound MeSC-04. The image was created with the PyMOL program [25].

For each program, the first 10 clusters were chosen based on the docking score and were subjected to a consensus docking methodology to obtain clusters populated by poses generated by different programs with root-mean-square deviation (RMSD) values just below 2.0 Å. Hierarchical clustering using the rms_analysis tool of the Gold suite showed four clusters at site A, while none clusters were revealed at site B. As a result, it was assumed that MeSC-04 can bind only site A on α-Syn fibrils. Subsequently, MM-GBSA free energy calculations [34] were used to obtain quantitative estimation of the binding free energies of the different MeSC-04 poses achieved from the molecular docking studies. The pose with the lowest free energy value was selected to evaluate protein–ligand interactions through Maestro and is displayed in Figure 7.

In detail, MeSC-04 bound to site A via Van der Waals and hydrogen bonding interactions with residues from Ala53 to Val74. MESC-04 showed the formation of hydrogen bonding interactions between the Gly73 of chain A and the tetrazole ring and a hydrogen bond between the Thr59 of chain C and a nitro group. Finally, further Van der Waals interactions were observed for the amino acids Ala53, Val55, Thr59, Glu61, Gly73, and Val74 (Figure 7). Interestingly, these data were consistent with the network of interactions that were suggested for SC-D docked into the NAC domain of α-Syn fibrils, as indicated in previous studies [13].

## 3. Materials and Methods

### 3.1. Ligand-Based Virtual Screening

Ligand-based virtual screening (LBVS) was carried out using the similarity-based search web tool SwissSimilarity (http://www.swisssimilarity.ch, accessed on 1 February 2021) [19] to screen molecules from the SPECS database (https://www.specs.net, accessed on 1 March 2021). Three different methods were independently employed to evaluate the similarity between the query molecules and those contained in the search database: FP2 fingerprints, Electroshape, and spectrophore. While the former is a 2D approach, the latter relies on the 3D structures of the molecules. The hits obtained by the LBVS runs were ranked according to a similarity score corresponding to the Tanimoto score for the FP2 fingerprints and to the Manhattan-based distance for the spectrophore and Electroshape approaches. Both scores can assume values between 0 and 1. A value of 0 indicates totally dissimilar molecules, while a value equal to 1 implies identical compounds.

### 3.2. In Vitro Testing (Th-T Fluorescence, Light Scattering, and Electron Microscopy Assays)

Human wt α-Syn was expressed and purified as previously reported [10,12,13,17]. For storage, the protein was lyophilized for 48 h and kept at −80 °C until use. When needed, the protein was carefully resuspended in sterile PBS 1X and filtered through a 0.22 µm filter to eliminate small aggregates and impurities. The in vitro aggregation of α-Syn in the presence and absence of the different compounds was performed as in previous studies [10,12,13,17]. Briefly, soluble α-Syn was placed in a sealed 96-well plate with each well containing 70 µM α-Syn, 40 µM Th-T in PBS 1X, a 1/8″ diameter Teflon polyball (Polysciences Europe GmbH, Eppelheim, Germany), and 100 µM concentrations of the different compounds in a final volume of 150 µL. For compound MeSC-04 the in vitro aggregation of α-Syn was measured at different doses, and the results are displayed in Figure S3 (see Supporting Material). As control samples, DMSO was added at the same final concentration as the treated samples. The plates were then incubated at 37 °C and 100 rpm in a Max-Q 4000 orbital shaker (ThermoScientific, Waltham, MA, USA). The Th-T fluorescence emission was measured every 2 h by exciting through a 430–450 nm filter and collecting with a 480–510 nm filter in a TECAN SPARK-1 plate reader (Tecan Trading AG, Männedorf, Switzerland). A light scattering analysis was performed in a Cary Eclipse Fluorescence Spectrophotometer (Agilent, Santa Clara, CA, USA) using final point samples. As previously described [10,13,17], 80 µL of the obtained mature aggregates were placed into a quartz cuvette and excited at 300 and 340 nm. Then, we collected the orthogonal emission at 280–320 or 320–360 nm, respectively. Each experiment was repeated at least in triplicate. A one-way ANOVA test with Dunnett’s multiple comparison was performed to analyze the statistical significance. For the transmission electron microscopy analysis, end-point aggregated samples were collected, diluted to 1:10 in PBS 1X, and gently sonicated for 5 min at intensity 2 in an Ultrasonic Cleaner sonicator (VWR International) to prevent large agglomerations of fibrils. Next, 5 µL of each sample was deposited on top of carbon-coated copper grids and incubated for 1 min, and the excess liquid was removed with filter paper. The grids were washed twice in Mili-Q water, and the excess liquid was again removed with filter paper. Finally, the samples were negatively stained by depositing 5 µL of 2% (*w*/*v*) uranyl acetate on top of the grids, incubating for 1 min, and carefully removing the excess liquid with filter paper. The grids were air-dried for at least 10 min and were observed using a Jeol JEM-1400 transmission electron microscope operating at an accelerating voltage of 120 kV. Representative images were obtained at a 2000× magnification, screening a minimum of 30 fields per sample.

### 3.3. Analysis of Potential Binding Sites

The analysis of putative ligand binding sites was performed on two α-Syn 3D structures retrieved from the RCSB Protein Data Bank (PDB codes 2N0A and 6FLT). The analysis of protein PDB 2N0A was conducted for one monomer by selecting only the chains A, B, C, D, and E, whereas we used the chains A, C, E, G, and I for the monomer of protein PDB 6FLT. Since PDB 6FLT does not encompass the complete protein, only the residues between Leu38 and Val95, the chains of PDB 2N0A were modified by removing the residues belonging to the flexible loops and preserving only the amino acids between Leu38 and Val95 using the software Pymol (https:///pymol.org, accessed on 25 February 2022). In this way, we were able to perform the alignment of 6FLT on 2N0A proteins in order to obtain overlapping results through the software Pymol. To obtain information about the flexibility of the selected structures, the RMSD values were calculated by Maestro 2021-4, considering the C-alpha compared with the 2N0A reference protein structure. Therefore, the RMSD value of 5.89 Å was obtained for 6FLT.

To probe the presence of different binding sites on the α-Syn fibrils, three programs, FTMap, fPocket, and SiteMap, were used.

The FTMap web server (https://ftmap.bu.edu, accessed on 11 January 2022) is an open-source mapping server that provides direct information about binding hot spots, druggability, and fragment-based drug discovery. The method distributes 16 small organic probe molecules of various shapes, sizes, and polarities on a macromolecule surface to define hot spots through conformational and spatial searches, clustering procedures, and an evaluation of the probe’s interaction energy over a dense grid using an empirical energy function including a continuum electrostatic term. Hot spots are smaller regions of proteins that are capable of contributing significantly to the binding of a drug to the binding site, and their strengths describe the druggability of the sites [35].

SiteMap is a module implemented in Schrodinger that is capable of identifying potential binding sites and predicting their druggability. SiteMap uses the interaction energies between the grid probes and the protein to search for favorable binding sites. To classify each site, there is a series of physical descriptors that are used. These include (i) the size of the site calculated from the number of site points, (ii) the degree of closure by the protein, (iii) solvent exposure, (iv) the tightness between the site points and the protein, (v) the hydrophobic and hydrophilic character of the site, and (vi) the ability with which a ligand can donate or accept hydrogen bonds. Finally, an overall SiteScore is calculated using a linear combination of terms based on the above factors [36].

fPocket (version 2.0) is an open-source program that is capable of detecting potential binding sites using Voronoi tessellation and sequential clustering steps. The method employs α-spheres to analyze the protein surface. An α-sphere is a sphere that is in contact with four atoms on its boundary and contains no inner atom inside. For a protein, very small spheres are found on the inside of the protein, while large spheres are found on the outside. Clefts and cavities coincide with spheres of intermediate radii [37]. fpocket is based on three main steps. During the first step, the whole set of alpha spheres is determined from the protein structure, and a prefiltered set of spheres is obtained. The second step involves identifying groups of spheres close to each other to identify pockets and remove all clusters of low interest. The last step involves estimating the properties of pocket atoms for the purpose of scoring each pocket [38].

The fPocket, SiteMap and FTMap software programs were used to evaluate the druggability of α-Syn fibrils and predict putative binding sites. To start with our computational studies, we analyzed two structures of α-Syn fibrils that are available in the RCSB PDB databases (PDB codes: 2N0A and 6FLT). The “hot spots” residues were identified for each prediction performed with the tools described above and were displayed on Maestro GUI [39]. The overlapping hot spots were merged by visual inspection. The obtained distinct sites are highlighted with different colors and displayed in Figure S1 (see Supporting Material).

The analyses performed with FTMap detected 15 cross clusters that were grouped at four different binding sites on 2N0A, whereas 11 clusters belonging to a single plausible binding site were detected for 6FLT. SiteMap identified five different binding sites for each protein. Finally, based on the fPocket analysis, we identified eight putative binding sites on 2N0A and two plausible binding pockets on 6FLT (see Figure S1 in the Supporting Material). Combining the results obtained from the application of the three distinct procedures, we identified two putative binding sites that could be druggable: in detail, site A (magenta), as the result of all three programs, and site B (green), as the result of SiteMap and fPocket. Although SiteMap and fPocket use completely different approaches, they showed comparable results in the classification of the binding sites.

### 3.4. Molecular Docking Studies

The binding mode of MeSC-04 was investigated using three different programs employing the 3D coordinates of the NMR structure of the α-syn fibrils (PDB code 2N0A). In this study, docking procedures were carried out by means of Autodock 1.5.7, Glide with the extra precision method (XP), and GOLD with the ChemScore fitness function. In addition, in the analysis conducted through GOLD, the “allow early termination” command was deselected, and the docking poses with RMSD values lower than 0.75 Å were grouped together. All setting parameters were kept by default. For all the docking protocols, the binding cavity was defined to include all residues within 10 Å of the center. For each site, centroid coordinates were calculated from fPocket outputs using Discovery Studio Visualizer. In detail, the centroid of coordinates x = 115.647963, y = 142.554128, and z = −35.965229 were used to define site A, while a box with the center coordinates x=92.100174, y = 140.774522, and z = −19.706261 was used to delineate site B.

The structure of MeSC-04 was built and minimized according to the Ammp calculation method implemented in Vega ZZ [40] and was subjected to 100 runs in each molecular docking run.

The 10 top-ranked docking poses obtained from the three different docking programs were selected and subjected to a hierarchical clustering procedure. The RMSD value of each docking pose compared with the others was calculated using an rms analysis software from the Gold suite. The group average method was used as a hierarchical clustering algorithm, and the poses were considered similar if they had an RMSD value less than 2.0 Å. Therefore, only clusters populated by poses generated by at least two molecular docking programs were selected. Subsequently, the poses belonging to the most populated cluster were submitted to a binding free energy calculation by the molecular mechanics–generalized born surface area (MM-GBSA) computational method, which was performed using the Prime MM-GBSA module of the Schrödinger suite. This method combines molecular mechanics energy and continuum solvation models. Although the MM/GBSA method cannot predict experimental binding free energies with absolute accuracy, it is a very efficient method for producing a good ranking of experimental values [34]. In this analysis, the VSGB solvation model and OPLS-4 as force field were used to perform MM-GBSA calculations.

## 4. Conclusions

We have disclosed MeSC-04 as a potent in vitro inhibitor of α-Syn amyloid formation; this compound might serve as a chemical template to design a new potential agent for the development of new therapeutics for PD. In detail, MeSC-04 demonstrated a similar level of activity with respect to SC-D in the Th-T fluorescence test as well as in the electron microscopy analysis. These data might be useful to confirm the role of the α-Syn NAC domain in controlling fibrillization. Based on the consideration that MeSC-04 contains a very simple chemical scaffold, we believe that this new chemotype might be easily functionalized to generate a large class of chemical congeners, thus improving our knowledge about structure–activity relationship information that controls the NAC domain recognition process. Moreover, the structural modifications could be addressed to improve the physicochemical properties to enhance the bioavailability in the central nervous system.

## Figures and Tables

**Table 1:** Number of hits obtained from the VS runs employing SC-D and ZPD-2 as query molecules. The number of compounds selected from each approach for further analysis is reported in brackets.

Query Cmpds	FP2	Electroshape ^a^	Spectrophores ^b^
SC-D	3 (3)	33 (33)	400 (108)
ZPD-2	2 (2)	400 (178)	400 (39)

^a^ Reference [21]; ^b^ Reference [22].

**Table 2:** List of the protein residues (in different chains) forming the identified binding sites, including residues within 15 Å of any centroid of the Fpocket results for protein 2N0A.

Putative Binding Sites	Residues
SITE A	VAL52A; ALA53A; THR54A; VAL55A; ALA56A; GLU57A; LYS58A; THR59A; LYS60A; GLU61A; VAL70A; VAL71A; THR72A; GLY73A; VAL74A; THR75A; THR92A; GLY51B; VAL52B; ALA53B; THR54B; VAL55B; ALA56B; GLU57B; LYS58B; THR59B; LYS60B; GLU61B; GLN62B; THR64B; VAL66B; VAL70B; VAL71B; THR72B; GLY73B; VAL74B; THR75B; ALA76B; THR92B; HIS50C; GLY51C; VAL52C; ALA53C; THR54C; VAL55C; ALA56C; GLU57C; LYS58C; THR59C; LYS60C; GLU61C; GLN62C; THR54C; VAL66C; ALA69C; VAL70C; VAL71C; THR72C; GLY73C; VAL74C; THR75C; ALA76C; THR92C; GLY93C; GLY51D; VAL52D; ALA53D; THR54D; VAL55D; ALA56D; GLU57D; LYS58D; THR59D; LYS60D; GLU61D; GLN62D; THR64D; VAL70D; VAL71D; THR72D; GLY73D; VAL74D; THR75D; ALA76D; THR92D; GLY51E; VAL52E; ALA53E; THR54E; VAL55E; ALA56E; GLU57E; LYS58E; THR59E; LYS60E; GLU61E; VAL70E; VAL71E; THR72E; GLY73E; VAL74E; THR75E
SITE B	VAL40A; GLY41A; SER42A; LYS43A; THR44A; LYS45A; GLU46A; VAL48A; TYR39B; VAL40B; GLY41B; SER42B; LYS43B; THR44B; LYS45B; GLU46B; GLY47B; VAL48B; VAL49B; HIS50B; TYR39C; VAL40C; GLY41C; SER42C; LYS43C; THR44C; LYS45C; GLU46C; GLY47C; VAL48C; VAL49C; HIS50C; TYR39D; VAL40D; GLY41D; SER42D; LYS43D; THR44D; LYS45D; GLU46D; GLY47D; VAL48D; VAL49D; HIS50D; GLY41E; SER42E; LYS43E; THR44E; LYS45E; GLU46E; VAL48E; VAL49E; HIS50E.

## Data Availability

Not applicable.

## Supplementary Materials

The following supporting information can be downloaded at: https://www.mdpi.com/article/10.3390/ijms232314844/s1.
