# Supplementary material for: Ligand-Based Discovery of a Small Molecule as Inhibitor of α-Synuclein Amyloid Formation
Source: Int J Mol Sci. 2022 Nov 27;23(23):14844. doi: 10.3390/ijms232314844 (PMC9738895; doi:10.3390/ijms232314844)
Supplement: Supplementary file 1 [file ijms-23-14844-s001.zip › ijms-1951624-supplementary.pdf]

Supplementary Material

Ligand-Based Discovery of a Small Molecule as Inhibitor of α-Synuclein Amyloid Formation

De Luca L. [1], Vittorio S.[1], Peña-Díaz S [2,3], Pitasi G.[1], Fornt-Suñé M. [2,3], Bucolo F.[1], Ventura S [2,3,4,] Gitto R, [1,*]

[1] Department of Chemical, Biological, Pharmaceutical and Environmental Sciences, University of Messina, Viale F. Stagno D'Alcontres 31, I-98166 Messina, Italy;

[2] Institut de Biotecnologia i Biomedicina, Universitat Autonoma de Barcelona, 08193 Bellaterra, Spain Affiliation;

[3] Departament de Bioquimica i Biologia Molecular, Universitat Autonoma de Barcelona, 08193 Bellaterra, Spain;

[4] ICREA, Passeig Lluis Companys 23, 08010 Barcelona, Spain

Corresponding: rosaria.gitto@unime.it; Tel.: +39-090-676-6413

List of contents

- Figure S1: Analysis of potential binding sites:. results from fPocket, SiteMap and FTMap
- Ramachandran plot analysis: Table S1 List of the protein residues forming the identified binding sites defined to include residues within 10 Å of any centroid of Fpocket results for protein 2N0A
- Figure S2:Structural characterization: ^{1}H-NMR and ^{13}C-NMR spectra of compound MeSC-04
- Figure S3: Th-T fluorescence at the end point of the aggregation reaction of 70 µM α-Syn in the absence (black) or presence of different doses of MeSC-04

Analysis of potential binding sites

The fPocket, SiteMap and FTMap software programs were used to evaluate the druggability of -Syn fibrils for predicting putative binding sites. We analysed two structures of -Syn fibrils available in the RCSB PDB databases (PDB codes: 2N0A and 6FLT). The "hot spots" residues were displayed on Maestro GUI [1]. The overlapping hot spots were merged by visual inspection. The obtained distinct sites are highlighted with different colours and displayed in Figure S1.

PDB code: 2N0A	PDB code: 6FLT

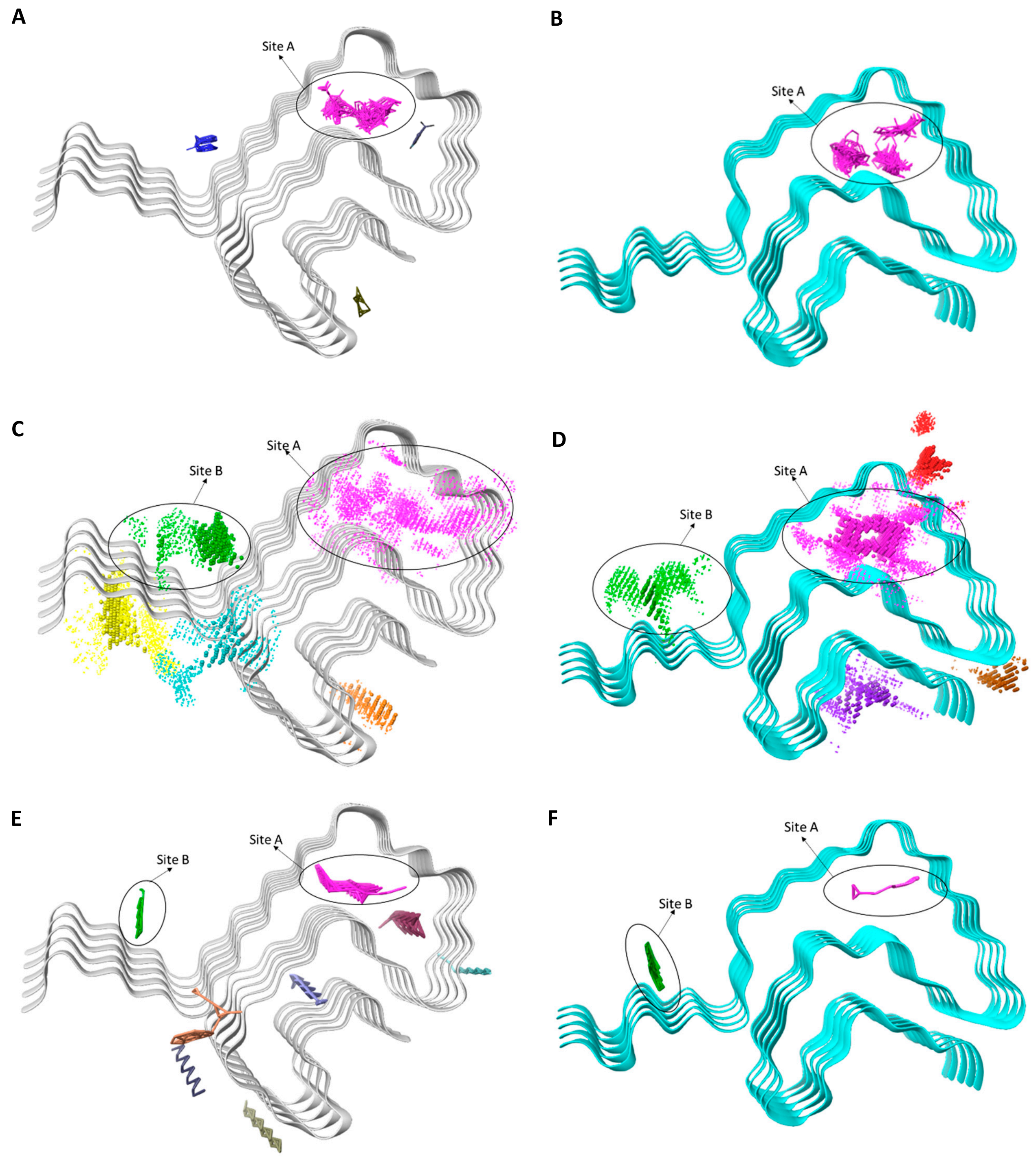

Figure S1. Structures of the α-Syn fibrils from 2N0A (platinum Cartoon) and from 6FLT (cyan Cartoon). The results of each analysis are reported as follows: (A) four binding sites detected by FTMap on 2N0A; (B) one

binding site found by FTMap on 6FLT; (C) five plausible binding sites found by sitemap on 2N0A; (D) five site bindings detected by sitemap on 6FLT; (E) eight binding sites identified by fPocket on 2N0A; (F) two binding sites found by fPocket on 6FLT. The identified plausible binding sites are displayed in magenta (site A) and green (site B).

Ramachandran plot analysis

According to the PROCHECK-Ramachandran plot analysis [2] the PDB 2N0A showed 85.1% of residues in the most favoured regions; 12.8% in the additional allowed regions and 2.1% in the generously allowed regions. Whereas PDB 6FLT showed 76.6% of residues in the most favoured regions and 23.4% in the additional permitted regions. Both PDBs showed no residue within the forbidden region. Based on these data, the docking studies were performed on the PDB 2N0A.

Table S1: PROCHECK-Ramachandran plot for α-synuclein fibrils from PDB 2N0A and 6FLT.

Protein	Ramachandran Plot	A	B	C
2N0A	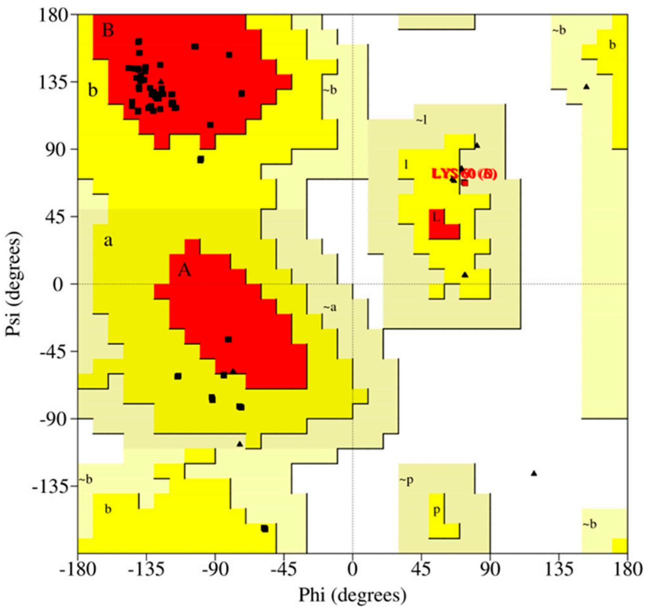	85.1%	12.8%	2.1%
6FLT	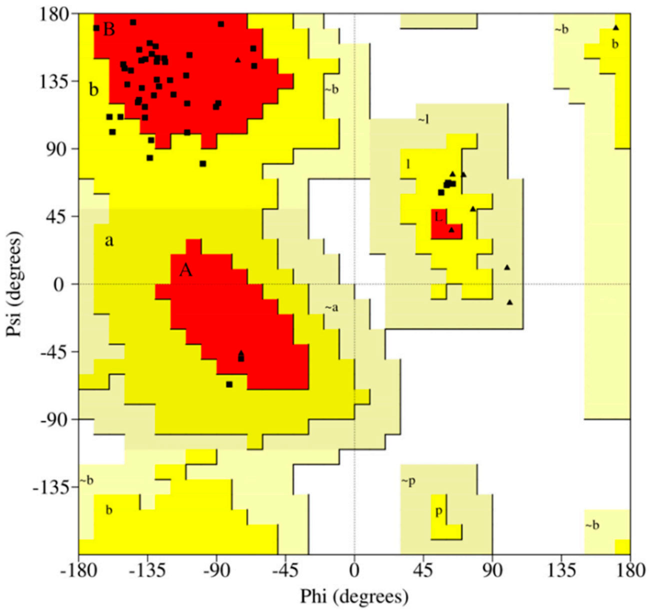	76.6%	23.4%	0%

A: % Residues in most favored regions [A,B,L]; B: % Residues in additional allowed regions [a,b,l,p]; C: % Residues in generously allowed regions [~a,~b,~l,~p]

Structural Characterization of compound MeSC-04

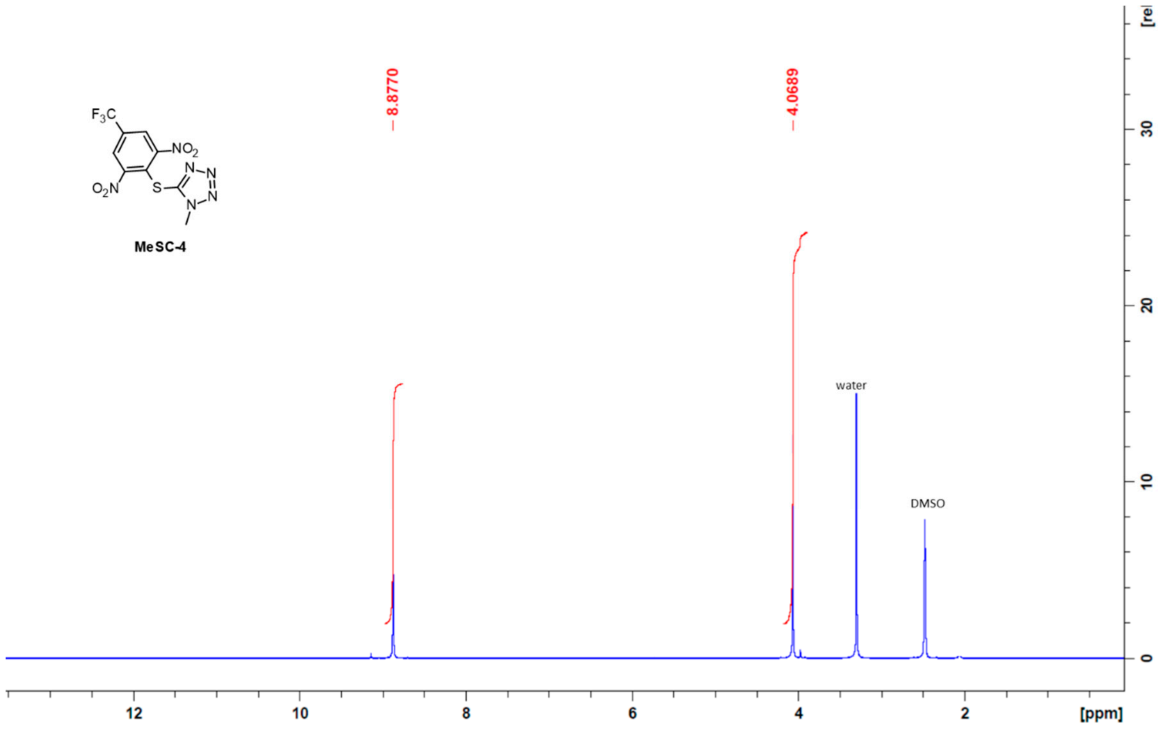

Figure S2a: ^{1}H-NMR (DMSO-d_6) spectrum of 5-(2,6-dinitro-4-(trifluoromethyl)benzyl)-1-methyl-1H-tetrazole

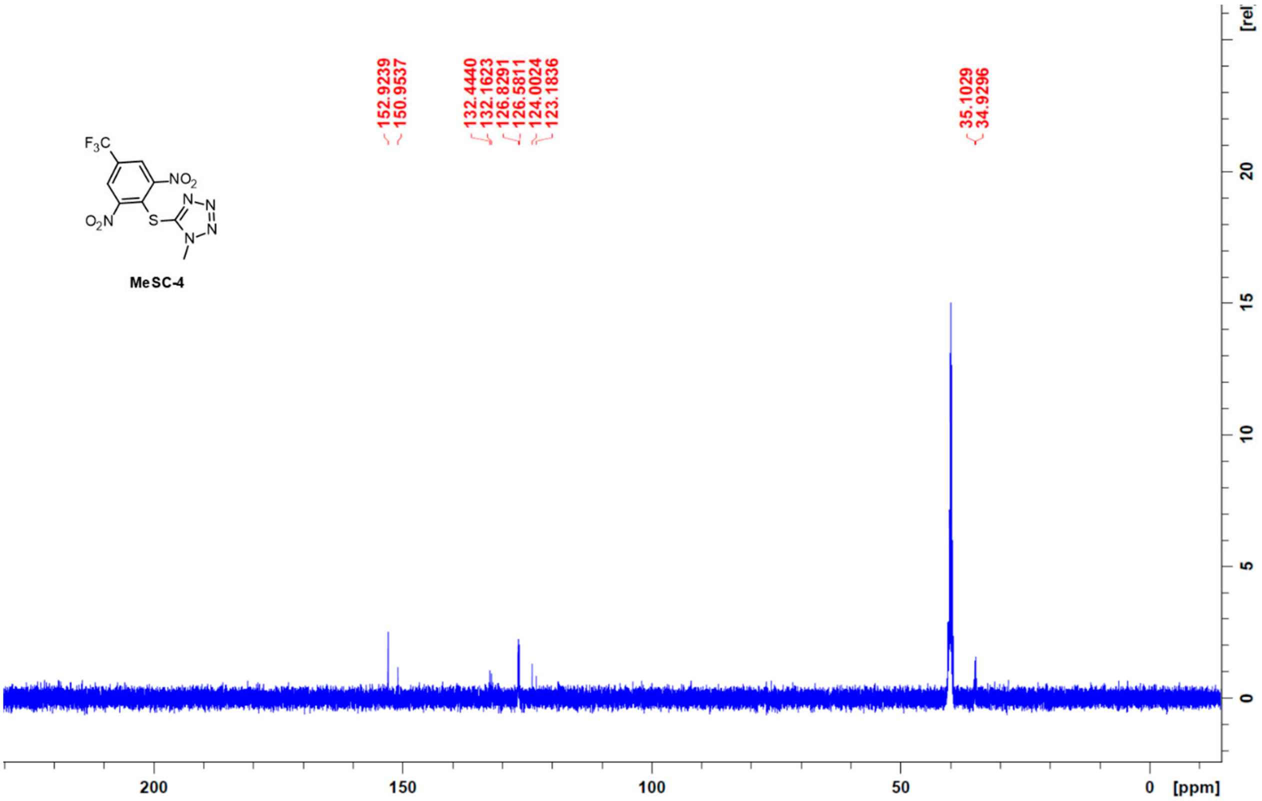

Figure S2b: ^{13}C-NMR (DMSO-d_6) spectrum of 5-(2,6-dinitro-4-(trifluoromethyl)benzyl)-1-methyl-1H-tetrazole

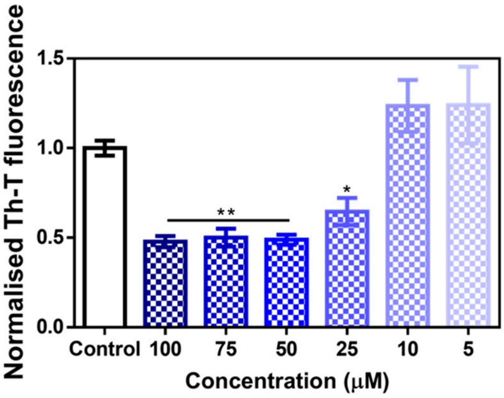

Figure S3. *In vitro* inhibitory characterization of compound MeSC-04. Normalised Th-T fluorescence at the end point of the aggregation reaction of 70 μM α-Syn in the absence (black) or presence of different doses (5, 10, 25, 60,75, 100 μM) of tested compound (colored in blue). The statistical significance was performed by one-way ANOVA test with Dunnett's multiple comparison (*p<0.05; **p<0.01; ***p<0.001). Th-T fluorescence is plotted as normalized means; error bars are represented as SE of mean values for N ≥ 3 in each experiment.

References

1. Schrödinger Release 2021-4: Maestro, S., LLC, New York, NY, 2021. .
2. Laskowski, R.A.; Macarthur, M.W.; Moss, D.S.; Thornton, J.M. Procheck - a Program to Check the Stereochemical Quality of Protein Structures. *J. Appl. Crystallogr.* **1993**, *26*, 283-291, doi:Doi 10.1107/S0021889892009944.
